# A systematic review of Amblyopia prevalence among the children of the world

**DOI:** 10.22336/rjo.2020.56

**Published:** 2020

**Authors:** Ali Mostafaie, Morteza Ghojazadeh, Hossein Hosseinifard, Hesam Manaflouyan, Fereshteh Farhadi, Nazli Taheri, Fariba Pashazadeh

**Affiliations:** * Department of Ophthalmology, Nikookari Ophthalmology University Hospital, Tabriz University of Medical Sciences, Tabriz, Iran; ** Research Center for Evidence based-medicine, Faculty of Medicine, Tabriz University of Medical Sciences, Tabriz, Iran; *** Student’s Committee, Research Center for Evidence based-medicine, Faculty of Medicine, Tabriz University of Medical Sciences, Tabriz, Iran

**Keywords:** Amblyopia, prevalence, child, systematic review, meta-analysis

## Abstract

**Objective:** to assess the prevalence of Amblyopia disease in the children of the world.

**Methods:** In order to perform this systematic review, PICO was considered as the research question. Then, the preferred keywords were searched in Medline (via PubMed), Embase, Scopus, Web of Science, and ProQuest databases. The retrieved citations were reviewed by two independent inspectors in a three-step process in terms of the title, abstract, and full-text, based on the inclusion criteria. The studies included in the review were critically evaluated and then were extracted by two dependent expert reviewers. Finally, the prevalence of Amblyopia disease in the children of the world was pooled by meta-analysis CMA v.2 software. The heterogeneity of the selected studies was evaluated using I2 and chi-square. Also, subgroup-analysis was performed using designs and continents.

**Results:** Out of 952 retrieved citations, 131 studies were included. The total prevalence of Amblyopia in the children of the world was calculated to be 4.3% [Pooled Prevalence: 4.3%, 95% CI: 2.6%-7.00%, P-value 0.0001]. In addition, the heterogeneity of the studies was reported to be high (Q: 48281.18, df: 56, p-value 0.001, I-square: 99.88). The subgroup analysis showed that America had the highest (5.57%, 95% CI: 2.23%-13.94%, P-value 0.0001) prevalence, and the lowest prevalence of Amblyopia in the children of the world was seen in Africa (7.1%, 95% CI: 0.003%-172.53%, P-value 0.05).

**Conclusion:** It can be concluded that the total prevalence of Amblyopia is 3.4%, but this estimate varies in all continents, especially in Africa. The major reason for this variation was reported to be the heterogeneity of studies. These assessments have investigated different populations in terms of severity of illness, age, and gender. Therefore, further worldwide high-quality and valid studies should be carried out to allow the calculation of the real prevalence of Amblyopia among children of the world.

**Abbreviations:** VA = visual acuity, ALSPAC = Avon Longitudinal Study of Parents and Children, JBI = Joanna Briggs Institute, PRISMA = Systematic Review and Meta-analysis, CMA = Comprehensive Meta-analysis Software

## Introduction

One of the most common disabilities among children is visual impairment [**1**]. Being able to manage this condition is a primary focus of the World Health Organization’s VISION 2020 campaign entitled “The Right to Sight” [**2**]. Many children of school age suffer from visual disorders that are delayed to be diagnosed prior to school entry. Visual disorders frequently lead to a number of Psychiatric disorders such as ADHD, impairments in learning, and dyslexia [**3**].

Amblyopia is considered as one of the most prominent causes of visual impairment in children and adult population [**4**-**6**]. It is defined as a decrease in visual acuity (VA) in one or both eyes [**7**], which is not rapidly recovered by refractive correction [**8**]. The children experiencing Amblyopia usually have a decreased quality of life compared to normal children. It is also responsible for poor academic performance [**9**,**10**]. Amblyopia is the most common visual problem in the children before school age. It is usually established in children up to the age of 7 or 8 [**11**]. Amblyopia will be fully cured if it is diagnosed and treated before the age of 9 or 10 [**12**,**13**]. However, if not diagnosed and treated correctly, it can result in a permanent visual disability [**14**].

Amblyopia is considered as one of the most prominent causes of visual impairment in children, juveniles, and elder populations [**4**-**6**]. Amblyopia is defined as an impairment of visual acuity (VA) in one or both eyes [**7**], which is not rapidly treated by the refractive correction method [**8**]. The children suffering from Amblyopia usually have less life quality than other children. In addition, Amblyopia has been reported to be responsible for poor performance in universities [**9**,**10**]. Amblyopia is the major reason for visual deficiency in children of pre-school age. It is often seen in children in the age group of up to 7 or 8 years [**11**]. Amblyopia can be completely healed in case of early diagnosis and treatment before the age of 9 or 10 [**12**,**13**]. However, the condition may lead to a visual disability for the rest of the life of the patient if not diagnosed and treated correctly [**14**]. Studies on Amblyopia indicate that refractive disorders are usual in all ages [**15**-**18**]. Nevertheless, a number of studies have evaluated the prevalence of Amblyopia [**19**-**30**]. These studies have reported the disorder to be present in 2% to 3% of the public population [**31**-**33**]. The approximate prevalence of Amblyopia has been reported to be in a range of 0.3% [**34**] to 5% [**35**] in pre-school children. During a school-study in Tanzania, the prevalence of the disorder has been reported to be 0.2% in children of 7-19 years of age [**30**]. During another study in Australia, it was found that the prevalence of the disorder among 6-year-old children was 1.8% [**25**]. Avon Longitudinal Study of Parents and Children (ALSPAC) was conducted in Britain to calculate the Prevalence of Amblyopia disorder among 7-year-old children. The results indicated that the prevalence of the disease among the study group was 3.6% [**28**]. Nonetheless, a number of clinical samples have reported a higher prevalence [**36**].

Evaluation of Amblyopia prevalence is crucial for clinicians and health policy decision-makers. They can plan on screening, detection, and intervention programs using this information. As described above, several kinds of research have evaluated the prevalence of Amblyopia in different populations and age ranges. It is obvious that an organized, precise, and categorized estimation of the prevalence of Amblyopia is necessary for physicians and healthcare decision-makers. We conducted a search in PubMed, Joanna Briggs Institute (JBI) database of systematic reviews and implementation reports, and PROSPERO, to find systematic reviews and meta-analysis that pooled and presented the Amblyopia prevalence in the world, however, no result was obtained. Thus, we systematically reviewed the diverse prevalence studies up to 2018. This review compared and categorized the studies that investigated the prevalence of Amblyopia in distinct populations and groups.

**Review question**

The aim of this study was to assess the prevalence of Amblyopia among children of the world.

**Inclusion criteria**

***Condition***

This study consisted of articles that included children diagnosed with Amblyopia. The most common etiology of these patients was being affected by strabismus or refractive disorders in their eyes. Children whose problem originated from eye trauma or congenital cataract were excluded.

***Context***

This study included articles that evaluated the prevalence of Amblyopia worldwide, which were performed by a pre-school screening program or by referring to medical centers and clinics.

***Participants***

Studies evaluating children who had an eye examination.

***Types of studies***

This study considered observational studies including prospective and retrospective cohort reviews, as well as a number of analytical and descriptive cross-sectional studies. In addition, several studies assessing data using experimental and quasi-experimental methods such as randomized controlled trials, non-randomized controlled trials, etc., which had assessed Amblyopia prevalence at the baseline, were included. Articles that have been published in any language from 1963 to June 2019 were included in this project.

## Materials and Methods

This systematic review was performed in accordance with the JBI methodology for systematic reviews of Prevalence studies [**37**], Preferred Reporting in Systematic Review and Meta-analysis (PRISMA) statement.

**Search strategy**

A three-stage search strategy was used in this study. An initial search limited to Medline (PubMed) database was performed, and after that, an analysis was conducted for the words that were included in the title and abstract sections. In addition, the index terms were used to describe the articles. Then, a second search was conducted, in which we used all detected keywords and index terms. This search was conducted in June 2019 across the following databases: Medline (via PubMed), Embase, Scopus, and Web of Science. In addition, the search for unpublished studies and gray literature included ProQuest (dissertation and thesis), and Google scholar. Eventually, the lists of all references of all reports and articles selected for critical appraisal were once again searched to look for any additional data. The whole search strategy for the Embase database is provided in **Appendix I**.

**Appendix I:** Search strategy Search strategy in Embase conducted in June 2019

Search	Query	Records retrieved
#1	'Amblyopia'/exp	10,159
#2	'Amblyopia':ti,ab	7,835
#3	'prevalence'/exp	586,022
#4	'prevalence':ab,ti	703,918
#5	#1 OR #2	11,446
#6	#3 OR #4	854,085
#7	#5 AND #6	1,058
#8	#7AND ([child]/lim OR [fetus]/lim OR [infant]/lim OR [newborn]/lim OR [preschool]/lim OR [school]/lim) AND ('Article'/it OR 'Article in Press'/it OR 'Conference Abstract'/it)	96

**Study selection**

After conducting the search, all citations were loaded into Endnote X7 software, and duplicates were deleted. Titles and abstracts were reviewed by two independent colleagues for evaluation of the inclusion criteria for the review. The full text of potentially eligible studies was obtained and evaluated thoroughly against the inclusion criteria by two independent reviewers. Full-text studies that were not in line with the inclusion criteria were omitted. Any disagreements between the reviewers were discussed and resolved or were commented by a third reviewer.

**Assessment of methodological quality**

Eligible studies were critically appraised by two independent reviewers at the study level using a standardized critical appraisal instrument from the Joanna Briggs Institute for prevalence studies. Any disagreements that arose between the reviewers were resolved through discussion, or with a third reviewer.

**Data extraction**

Two independent reviewers extracted data from the articles, using the modified standardized JBI data extraction tool. The extracted data consisted of specific details on the authors, publication year, populations (age), study setting and country, prevalence of Amblyopia. Any disagreements that arose between the reviewers were resolved through discussion, or with a third reviewer. The authors of the articles were contacted for obtaining any missing data, as well as any needed additional data.

**Data synthesis**

Data were pooled using a statistical meta-analysis with comprehensive Meta-analysis Software (CMA) v5.2 Software. For analysis, effect sizes were detailed in proportion with a confidence interval of 95% and were calculated. Heterogeneity was assessed using the standard chi-squared and I2 tests. Statistical analyses were performed using random effect method [**38**]. Analyses of subgroups were performed according to their continents. As there were 10 or more studies included in the meta-analysis, a funnel plot was generated in CMA v5.2 to assess the bias of publication. Statistical tests for the asymmetry of funnel plot such as the Egger test, Begg test, and Harbord test were conducted as needed.

## Results

**Study inclusion**

**Fig. 1:**
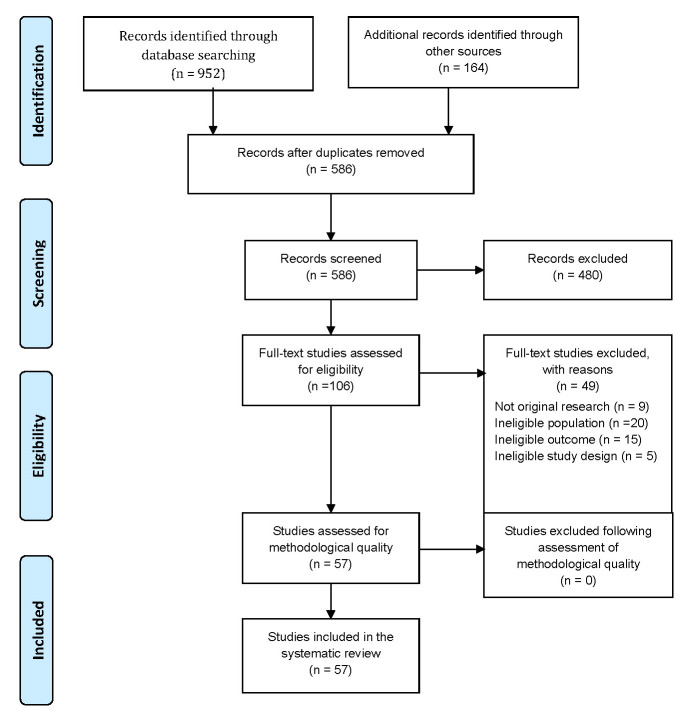
Search results and study selection and inclusion process [**43**]

Studies were identified by a comprehensive search in which 131 papers were selected according to title and abstract screening. In the next step, 57 studies were selected for full-text reviewing in which 57 studies were critically appraised. The process of step-by-step selection and also the reasons for exclusion of the studies in the full-text selection step are presented in the PRISMA flowchart (**Fig. 1**).

**Methodological quality**

The critical appraisal was conducted on 57 studies that were selected for this step. Overall, the quality of these articles was reported to be high. Hence, all studies were included in data extraction and synthesis. The results of the appraisal are presented in **Appendix II**.

**Appendix II:** Critical appraisal results of eligible studies

Study	Q1	Q2	Q3	Q4	Q5	Q6	Q7	Q8	Q9
Beck R (2002)	Y	Y	Y	Y	Y	Y	Y	Y	Y
Repka M et al. (2010)	NA	NA	NA	Y	NA	Y	Y	Y	Y
Zhao J et al. (2000)	NA	NA	NA	Y	NA	Y	Y	Y	Y
Thompson JR et al. (1991)	Y	Y	Y	Y	Y	Y	Y	Y	Y
Tarczy-Hornoch K et al. (2009)	NA	NA	NA	Y	NA	Y	Y	Y	Y
Tarczy-Hornoch K et al. (2013)	NA	NA	NA	Y	NA	Y	Y	Y	Y
Tarczy-Hornoch K et al. (2007)	NA	NA	NA	Y	NA	Y	Y	Y	Y
McKean-Cowdin R et al. (2013)	NA	NA	NA	Y	NA	Y	Y	Y	Y
Klimek DL et al. (2004)	Y	Y	Y	Y	Y	Y	Y	Y	Y
Unsal A et al. (2009)	NA	NA	NA	Y	NA	Y	Y	Y	Y
Marasini S et al. (2010)	NA	NA	NA	Y	NA	Y	Y	Y	Y
Fu J et al. (2014a)	NA	NA	NA	Y	NA	Y	Y	Y	Y
Fu J et al. (2014b)	NA	NA	NA	Y	NA	Y	Y	Y	Y
Fotouhi A et al. (2004)	NA	NA	NA	Y	NA	Y	Y	Y	Y
FitzGerald D et al. (2005)	Y	Y	Y	Y	Y	Y	Y	Y	Y
Khandekar R et al. (2009)	NA	NA	NA	Y	NA	Y	Y	Y	Y
Fan D et al. (2011)	NA	NA	NA	Y	NA	Y	Y	Y	Y
Gronlund MA (2006)	Y	Y	Y	Y	Y	Y	Y	Y	Y
Pi LH et al. (2012)	NA	NA	NA	Y	NA	Y	Y	Y	Y
Elflein H et al. (2015)	NA	NA	NA	Y	NA	Y	Y	Y	Y
Aldebasi YM (2015)	NA	NA	NA	Y	NA	Y	Y	Y	Y
Friedman DS et al. (2009)	NA	NA	NA	Y	NA	Y	Y	Y	Y
Ying G et al. (2014)	NA	NA	NA	Y	NA	Y	Y	Y	Y
Dray JP et al. (2002)	Y	Y	Y	Y	Y	Y	Y	Y	Y
Fiergang D et al. (1999)	Y	Y	Y	Y	Y	Y	Y	Y	Y
Bhandari G et al. (2015)	NA	NA	NA	Y	NA	Y	Y	Y	Y
Noche Ch et al. (2011)	Y	Y	Y	Y	Y	Y	Y	Y	Y
Wedner S et al. (2000)	NA	NA	NA	Y	NA	Y	Y	Y	Y
Ibrahim FM et al. (2013)	NA	NA	NA	Y	NA	Y	Y	Y	Y
Bandrakalli P et al (2012)	NA	NA	NA	Y	NA	Y	Y	Y	Y
Beckingsale PS et al. (2003)	Y	Y	Y	Y	Y	Y	Y	Y	Y
Donnelly UM et al. (2005)	NA	NA	NA	Y	NA	Y	Y	Y	Y
Høeg T et al. (2014)	NA	NA	NA	Y	NA	Y	Y	Y	Y
de Koning H et al. (2013)	Y	Y	Y	Y	Y	Y	Y	Y	Y
Lai YW et al. (2009)	Y	Y	Y	Y	Y	Y	Y	Y	Y
Matsuo T et al. (2005)	NA	NA	NA	Y	NA	Y	Y	Y	Y
Newman DK et al. (1996)	Y	Y	Y	Y	Y	Y	Y	Y	Y
Scott W et al. (2005)	Y	Y	Y	Y	Y	Y	Y	Y	Y
Thapa R (2010)	Y	Y	Y	Y	Y	Y	Y	Y	Y
KAREN HENDLER et al. (2016)	NA	NA	NA	Y	NA	Y	Y	Y	Y
Mohammed Aftab Maqsud et al. (2015)	NA	NA	NA	Y	NA	Y	Y	Y	Y
Asem Hameed (2016)	NA	NA	NA	Y	NA	Y	Y	Y	Y
Chen X et al. (2015)	NA	NA	NA	Y	NA	Y	Y	Y	Y
Habib Ojaghi (2016)	NA	NA	NA	Y	NA	Y	Y	Y	Y
Bu-Dan Hu et al. (2015)	NA	NA	NA	Y	NA	Y	Y	Y	Y
Mezbah Uddin et al. (2016)	NA	NA	NA	Y	NA	Y	Y	Y	Y
Chun-Ling Zhang et al. (2016)	NA	NA	NA	Y	NA	Y	Y	Y	Y
Srijana Adhikari et al. (2015)	NA	NA	NA	Y	NA	Y	Y	Y	Y
Yousef Homood Aldebasi (2015)	NA	NA	NA	Y	NA	Y	Y	Y	Y
Serap Azizoğlu et al. (2017)	NA	NA	NA	Y	NA	Y	Y	Y	Y
Mladen Bušić et al. (2016)	NA	NA	NA	Y	NA	Y	Y	Y	Y
Stela P et al. (2015)	Y	Y	Y	Y	Y	Y	Y	Y	Y
Li-Li Sun et al. (2016)	NA	NA	NA	Y	NA	Y	Y	Y	Y
Riyad G. Banayot (2016)	Y	Y	Y	Y	Y	Y	Y	Y	Y
Griffith JF et al. (2015)	NA	NA	NA	Y	NA	Y	Y	Y	Y
AbbasAli Yekta et al. (2011)	NA	NA	NA	Y	NA	Y	Y	Y	Y
Rohit Varma et al. (2017)	NA	NA	NA	Y	NA	Y	Y	Y	Y
Seong Hun Jeong and Ungsoo Samuel Kim (2013)	NA	NA	NA	Y	NA	Y	Y	Y	Y
Eedy Mezer et al. (2017)	NA	NA	NA	Y	NA	Y	Y	Y	Y
Ou Xiao et al. (2015)	NA	NA	NA	Y	NA	Y	Y	Y	Y
abbasali Yekta et al. (2016)	NA	NA	NA	Y	NA	Y	Y	Y	Y
Total				100		100	100	100	100
Y = Yes, N = No, U = Unclear, NA = Not Applicable; JBI critical appraisal checklist for Studies Reporting Prevalence Data: Q1 = Was the sample frame appropriate to address the target population??; Q2 = Were study participants sampled in an appropriate way?; Q3 = Was the sample size adequate?; Q4 = Were the study subjects and the setting described in detail?; Q5 = Was the data analysis conducted with sufficient coverage of the identified sample?; Q6 = Were valid methods used for the identification of the condition?; Q7 = Was the condition measured in a standard, reliable way for all participants?; Q8 = Was there appropriate statistical analysis?; Q9 = Was the response rate adequate, and if not, was the low response rate managed appropriately?									

**Characteristics of included studies**

Out of 57 studies, five cohort studies, one clinical trial, one case series, and 50 cross-sectional studies were included. The details of these studies are presented in **Table 1**.

**Table 1:** The characteristics of included studies

Authors (Year)	Country	Study Design	Sample Size	Age Range/ Mean	Prevalence
Beck R (2002)	USA	Prospective cohort	175	97 ± 26 days	0.23
Repka M et al. (2010)	Maryland	Clinical trials	2635	<18 years	0.59
Zhao J et al. (2000)	China	Cross-sectional	5884	5-15 years	0.01
Tarczy-Hornoch K et al. (2009)	California	Cohort	1663	30 to 72 months	0.01
			1701		0.01
Tarczy-Hornoch K et al. (2013)	California	Cohort	939	30 months of age or older.	0.01
			947		0.01
McKean-Cowdin R et al. (2013)	California	Cross-sectional	9172	30 to 72 months	0.02
Klimek DL et al. (2004)	Missouri	Cross-sectional	418	Mean age: 5 years	0.09
Unsal A et al. (2009)	Turkey	Cross-sectional	1606	Mean age: 10.52 ± 2.28 years	0.05
Marasini S et al. (2010)	Nepal	Descriptive	1802	Mean age: 10.78 ± 3.61 years	0.00
Fu J et al. (2014a)	China	Cross-sectional	2893	Mean age: 7.1 ± 0.4	0.01
Fu J et al. (2014b)	China	Cross-sectional	2260	Mean age: 12.4 ± 0.6	0.03
Fotouhi A et al. (2004)	Iran	Cross-sectional	4565	1-70 Months	0.08
FitzGerald D et al. (2005)	USA	Cross-sectional	178		0.76
Khandekar R et al. (2009)	Iran	Cross-sectional	1400000	3-6 years	0.01
Fan D et al. (2011)	Hong Kong	Cross-sectional	1424		0.17
Gronlund MA (2006)	Sweden	Cross-sectional	143	4-15 years	0.04
Pi LH et al. (2012)	China	Cross-sectional	3079	6-15 years	0.02
Elflein H et al. (2015)	Germany	Prospective cohort	3227	35-74 Months	0.06
Aldebasi YM (2015)	Saudi Arabia	Cross-sectional	5176	6-13 years	0.04
Friedman DS et al. (2009)	USA	Cross-sectional	673		0.02
			873		0.01
Ying G et al. (2014)	USA	Cross-sectional	481	3-5 years	0.05
			2072		0.03
			343		0.03
			796		0.05
			145		0.03
Fiergang D et al. (1999)	USA	Cross-sectional	80		0.71
Bhandari G et al. (2015)	Nepal	Observational	8017	<16 years	0.01
Noche Ch et al. (2011)	Cameroon	Cross-sectional	314	5-15 years	0.09
Wedner S et al. (2000)	Tanzania	Cross-sectional	1386	7-19 years	0.00
Ibrahim FM et al. (2013)	Brazil	Cross-sectional	1590	10-15 years	0.06
Bandrakalli P et al. (2012)	Southern India	Cross-sectional	14423	1-15 years	0.05
Beckingsale PS et al. (2003)	Australia	Retrospective case series	28		0.39
Donnelly UM et al. (2005)	Ireland	Cross-sectional	1582		0.02
Høeg T et al. (2014)	Denmark	Cross-sectional	3826	<20 years	0.01
de Koning H et al. (2013)	Netherlands	Cohort	4624	7 years	0.03
Lai YW et al. (2009)	Taiwan	Cross-sectional	618	Mean age: 5.2 years	0.05
Matsuo T et al. (2005)	Japan	Cross-sectional	374		0.00
Thapa R (2010)	Nepal	Descriptive	78	Mean:16	0.19
KAREN HENDLER et al. (2016)	USA	Retrospective, cross-sectional	11260	3-5 years	0.09
Asem Hameed (2016)	Pakistan	Cross-sectional	1644	5-15 years	0.02
Chen X et al. (2015)	China	Cross-sectional, cohort	5667	Mean: 57.89 ± 8.573 months	0.01
Bu-Dan Hu et al. (2015)	China	Cross-sectional	600		0.04
Mezbah Uddin et al. (2016)	Bangladesh	Cross- sectional	900	Mean: 5. 47 ± 0. 64 years	0.11
Chun-Ling Zhang et al. (2016)	China	Cross-sectional	7291	3-7 years	0.32
Yousef Homood Aldebasi (2015)	Saudi Arabia	Cross- sectional	5176	Mean: 9.53 ± 1.88 years	0.04
Serap Azizoğlu et al. (2017)	Turkey	Cross-sectional	823	Mean: 6.7 ± 2.2 years	0.02
Mladen Bušić et al. (2016)	Croatia	Cross-sectional	15648	48-54 months	0.08
Stela P et al. (2015)	Bulgaria	Cross-sectional	285	4 to 18 Years	0.21
Li-Li Sun et al. (2016)	China	Cross-sectional	1170	3 to 6 years	0.43
Riyad G. Banayot (2016)	Palestine	Cross-sectional	887	Mean: 7.14 years	0.14
Griffith JF et al. (2015)	USA	Retrospective 12-year, cross-sectional	63,841	Mean: 6.27 ± 0.85 Y	0.01
AbbasAli Yekta et al. (2011)	Iran	Cross-sectional	1,551	Mean: 11.2 ± 2.4 years	0.02
Ou Xiao et al. (2015)	Multi country	Cross-sectional	39321	5 to 15 years	0.01
Abbasali Yekta et al. (2016)	Iran	Cross-sectional	1,130	Mean age: 11.05 ± 2.93 years	0.03

**Fig. 2:**
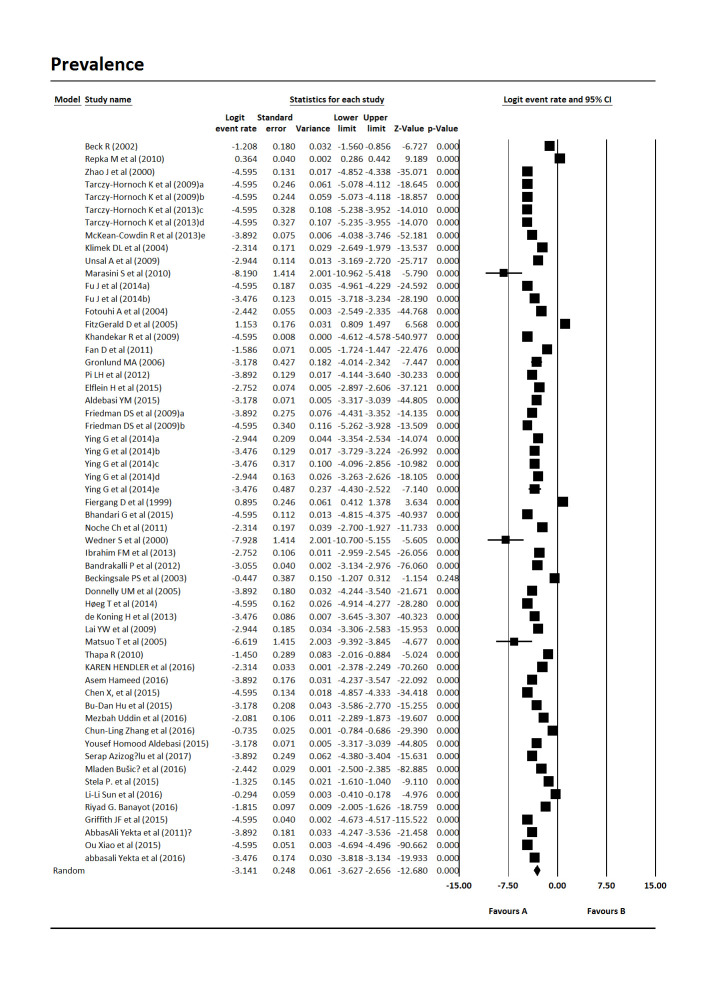
The total prevalence of Amblyopia in children worldwide

**Fig. 3:**
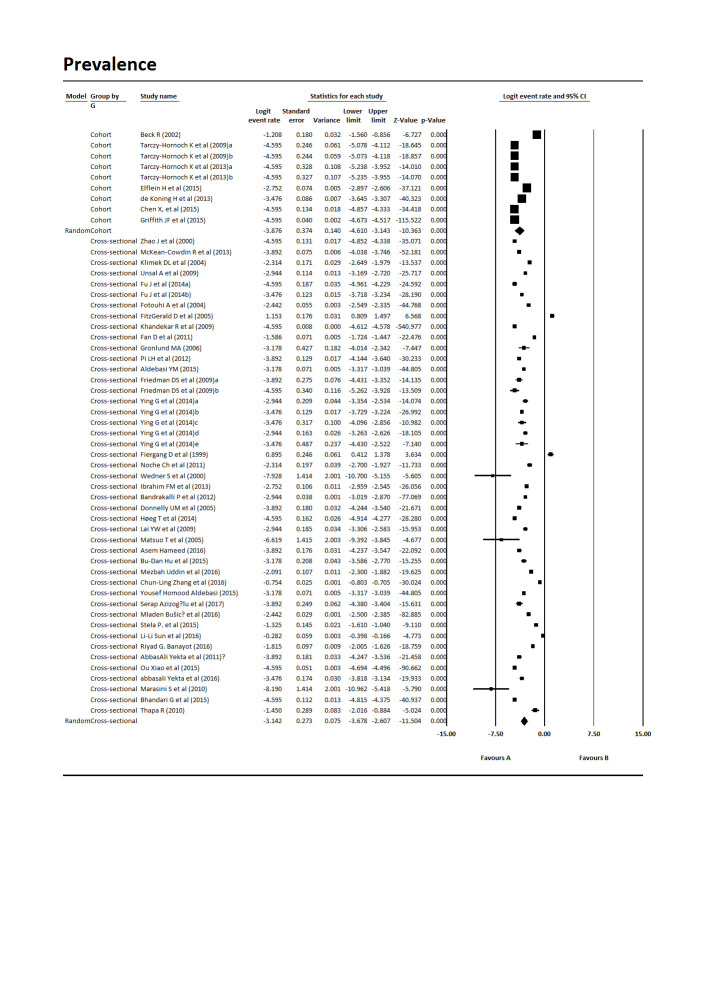
The prevalence of Amblyopia in children worldwide based on study design

**Fig. 4:**
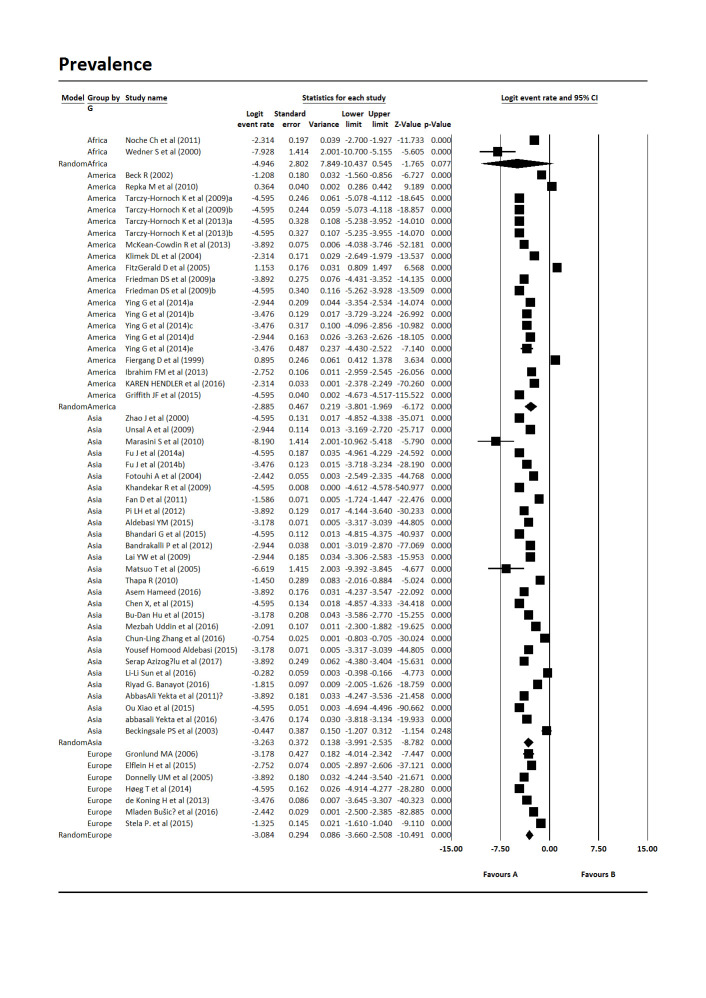
The prevalence of Amblyopia in children worldwide based on continent

The results from the meta-analysis of the data indicated that the total prevalence of Amblyopia in children worldwide is 4.3% [Pooled Prevalence = 4.3%, 95% CI:2.6%-7.00%, P-value 0.0001] (**Fig. 2**). The heterogeneity of included studies was very high (Q = 48281.18, df = 56, p-value 0.001, I-square = 99.88). The subgroup analysis of studies was carried out based on the design of the study and continent. The results showed that the prevalence of Amblyopia in the children of the world in cross-sectional studies was 4.31% [Pooled Prevalence = 4.31%, 95% CI:2.53%-7.37%, P-value 0.0001] and in cohort studies was 2.07% [Pooled Prevalence = 2.07%, 95% CI:1%-4.31%, P-value 0.0001] (**Fig. 3**). Based on the subgroup analysis according to continent, America (5.58%, 95% CI: 2.23%-13.94%, P-value 0.0001) had the highest prevalence and Europe (4.58%, 95% CI:2.57%-8.14%, P-value 0.0001), Asia (3.83%, 95% CI:1.85%-7.92%, P-value 0.0001) and Africa (0.71%, 95% CI:0.002%-172.46%, P-value 0.05) had the lowest prevalence of Amblyopia in children worldwide (**Fig. 4**).

## Discussion

Amblyopia is considered as one of the most common reasons for visual disorders in children and adult population. The prevalence of Amblyopia has been evaluated in numerous studies [**19**-**30**]. Preceding researches have reported rates varying from 2% to 3% [**31**-**33**]. The approximated prevalence of Amblyopia in children who have not entered school differs from 0.3% [**34**] to 5%. A number of projects have been tried to assess the prevalence of Amblyopia in diverse populations and age groups. The aim of this project was to systematically review the diverse prevalence articles undergone until 2018. This study compared and categorized the studies investigating the prevalence of Amblyopia in distinct populations and groups. The results from the meta-analysis indicated that the total prevalence of Amblyopia in children worldwide is 4.3%. According to the analysis of the subgroups based on the continent, America (5.57%) had the highest prevalence and Europe (4.57%), Asia (3.8%) and Africa (0.71%, 95% CI: 0.003%-172.53%, P-value 0.05) had the lowest prevalence of Amblyopia in children worldwide. There was a high report of incidence in one of the American studies, which was because of a low number of patients (seven) with unilateral or asymmetric congenital ptosis and compensatory head posturing [**39**]. The high incidence of Amblyopia in these patients may have occurred due to the absence of significant anisometropia and strabismus. This condition may be corrected using the compulsive examination and prophylactic part-time occlusion therapy. In another American study, a high prevalence of Amblyopia was reported, which was due to high myopia since children less than 10 years of age with high myopia have a high risk of having Amblyopia, strabismus, and anisometropia [**40**]. There was a direct relationship between myopia and Amblyopia. High myopes with anisometropia or strabismus had a relatively equal chance of Amblyopia.

Due to the low rate of studies in Africa (2 studies), the calculated prevalence was not significant and we could not find a low or high prevalence in this continent. More studies in this continent need to be done to estimate the prevalence and incidence of Amblyopia more precisely.

The heterogeneity of the included studies was very high, which may have been because of the real variation in the treatment outcomes and may include features of the population such as the severity of the disease, age, and gender [**41**]. Reducing heterogeneity between studies might be possible by using standardized measures to make data more meaningful in international comparisons. A number of other variables complicate the comparison process. These include deprivation, age of first treatment intervention, the onset of the condition, first optical correction, patients’ ages at the time of the study, variability in examiners’ findings, patient cooperation, compliance to treatment and wearing of spectacles, the interaction of other conflicting etiological elements, and minification due to optical correction [**40**].

Estimation of the prevalence of Amblyopia is important for both clinicians and health policy decision-makers for reaching an understanding of the need for screening, detection, and intervention in the community. A number of factors should be considered when deciding on introducing the eye screening programs in primary schools, including the prevalence and health, educational, or work impact of poor eyesight within the population, the human and financial resources available for screening, the cost and effectiveness of the screening and the treatment is given, and the availability and compliance with any treatment offered [**42**]. The limitation of this study was the low number of studies in some continents such as Africa, in which we were not able to make a real estimation of the prevalence. This continent seems to have a high prevalence of Amblyopia and because of low resources, the actual evaluation of the disease is yet to be extracted. However, because of the lack of well-designed research studies, poor standards of assessing Amblyopia, and insufficient treatment evaluation, the continued scarcity of robust evidence leaves gaps in knowledge that need to be addressed. The resources on the use of the existing policies should be re-allocated and revisited for the prevention and management of Amblyopia, in addition to considering the current disease burden.

## Conclusions

It can be stated that the total prevalence of Amblyopia is 3.4%. However, this estimation may vary in all continents especially for Africa. The major cause of this variation is the heterogeneity of studies that have investigated different populations in the severity of illness, age, and gender. Hence, high-quality and valid studies should be carried out in the world to find the real prevalence of Amblyopia among children.

**Conflict of Interest**

The authors state no conflict of interest.

**Acknowledgements**

None.

**Sources of Founding**

None.

**Disclosures**

None.
